# An Overview of Vaccines against SARS-CoV-2 in the COVID-19 Pandemic Era

**DOI:** 10.3390/pathogens10081030

**Published:** 2021-08-14

**Authors:** Alejandro Pascual-Iglesias, Javier Canton, Ana Maria Ortega-Prieto, Jose M. Jimenez-Guardeño, Jose Angel Regla-Nava

**Affiliations:** 1 The Innate Immune Response Group, IdiPAZ, La Paz University Hospital, 28046 Madrid, Spain; alejandro.pascual.iglesias@idipaz.es; 2 Tumor Immunology Laboratory, IdiPAZ, La Paz University Hospital, 28046 Madrid, Spain; 3 International Institute for Defense and Security (CISDE), 41007 Sevilla, Spain; jcanton@cisde.es; 4 Department of Infectious Diseases, School of Immunology & Microbial Sciences, King’s College London, London SE1 9RT, UK; Ana.ortega.prieto@kcl.ac.uk; 5 Center for Infectious Disease and Vaccine Research, La Jolla Institute for Immunology, La Jolla, CA 92037, USA; 6 Department of Microbiology and Pathology, University Center for Health Science (CUCS), University of Guadalajara, Guadalajara 44340, Mexico

**Keywords:** coronavirus, SARS-CoV-2, COVID-19, vaccine, clinical trial, pandemic

## Abstract

The emergence of SARS-CoV-2 in late 2019 led to the COVID-19 pandemic all over the world. When the virus was first isolated and its genome was sequenced in the early months of 2020, the efforts to develop a vaccine began. Based on prior well-known knowledge about coronavirus, the SARS-CoV-2 spike (S) protein was selected as the main target. Currently, more than one hundred vaccines are being investigated and several of them are already authorized by medical agencies. This review summarizes and compares the current knowledge about main approaches for vaccine development, focusing on those authorized and specifically their immunogenicity, efficacy preventing severe disease, adverse side effects, protection, and ability to cope with emergent SARS-CoV-2 variants.

## 1. Introduction

Coronaviruses (CoVs) are a wide group of single-stranded, positive-sense RNA viruses accountable for a broad range of emerging and existing diseases in human and domestic animals [1]. Historically, human coronaviruses have been associated with common cold symptoms in healthy individuals and relatively rare clinical illnesses in immunocompromised individuals, infants, and the elderly (human coronaviruses NL63, 229E, OC43, and HKU1) [1].

Bats of certain species are well recognized as being the natural host for a broad spectrum of CoVs. This provides an ideal scenario for recombination events between different viruses that infect bats, contributing to the emergence of new pathogenic viruses able to cross species barriers and cause pathogenic diseases in other humans or animals [2]. Indeed, since the beginning of this century, three previously unknown highly pathogenic human CoVs have crossed species barriers successfully from animal reservoirs to cause severe pneumonia in humans [3].

At the end of 2002, severe acute respiratory syndrome coronavirus 1 (SARS-CoV-1) emerged in Southeast China, infecting 8000 people globally with an average mortality rate of 10%. The SARS-CoV-1 global outbreak was brought under control in July 2003 and has not re-emerged since then. However, SARS-like coronaviruses closely related to SARS-CoV-1 are still present in bats identified worldwide, making another coronavirus outbreak a potential possibility [4]. Indeed, Middle East respiratory syndrome coronavirus (MERS-CoV), a novel human coronavirus, was identified in 2012 in Jeddah, Saudi Arabia, causing pneumonia and renal failure in infected patients [5]. Since then, MERS-CoV continues to circulate, infecting at least 2494 people, 858 cases of which have led to death, showing a mortality rate of 34% [6].

Furthermore, at the end of 2019, severe acute respiratory syndrome coronavirus 2 (SARS-CoV-2) was described as the etiological agent of the coronavirus disease 2019 (COVID-19) pandemic. SARS-CoV-2 emerged in China and rapidly spread on a global scale [7,8]. On 11 March 2020, the World Health Organization (WHO) declared the COVID-19 outbreak as a pandemic. As of July 2021, the number of SARS-CoV-2 identified infections, according to WHO, continues to rise with more than 190 million cases and a global death toll of more than 4 million people, especially in the elderly before the vaccination campaigns started [9]. COVID-19 deaths by race, ethnicity, age, health worker and territory are summarized by Howard [10]

All three highly pathogenic CoVs can cause acute respiratory distress syndrome (ARDS) and severe pneumonia. This leads to severe and life-threatening lung dysfunction, characterized by widespread inflammation in the lungs [11,12]. Moreover, SARS-CoV-2 is characterized as being more contagious than SARS-CoV-1 and MERS-CoV. This has severely impacted the global health, economy and also has put an unprecedented strain on health facilities worldwide. Furthermore, the lack of safe and effective antiviral drugs or treatments validated through large population studies worsens the situation, remaining a major threat to humankind and emphasizing the necessity for better control of SARS-CoV-2 infections.

Fortunately, effective and safe vaccines to prevent COVID-19 are available and represent an important tool to help contain the SARS-CoV-2 pandemic and preserve public health. Vaccination can reduce virus transmission and the severity of the disease, particularly in groups at risk for complications, including the elderly and people with underlying health conditions. Here, we review the different strategies and approaches for vaccine development against SARS-CoV-2.

## 2. Timelines for Vaccine Development

Vaccination has greatly contributed to human and animal global health, despite its undesirable side effects. This is particularly seen in the developing world, being considered one of the most important achievements in science and medicine. Furthermore, vaccines help mitigate the dispersion of infectious diseases and reduce the economic impact on healthcare systems [13,14].

The history of vaccines and immunization started in 1796 with Edward Jenner attempting to develop a vaccine against smallpox. To date, smallpox and rinderpest are the only human and animal infections that are completely eradicated. In 1980, the WHO declared smallpox eradicated [15] and rinderpest virus eradication was formally recognized in 2011 [16].

**Figure 1:**
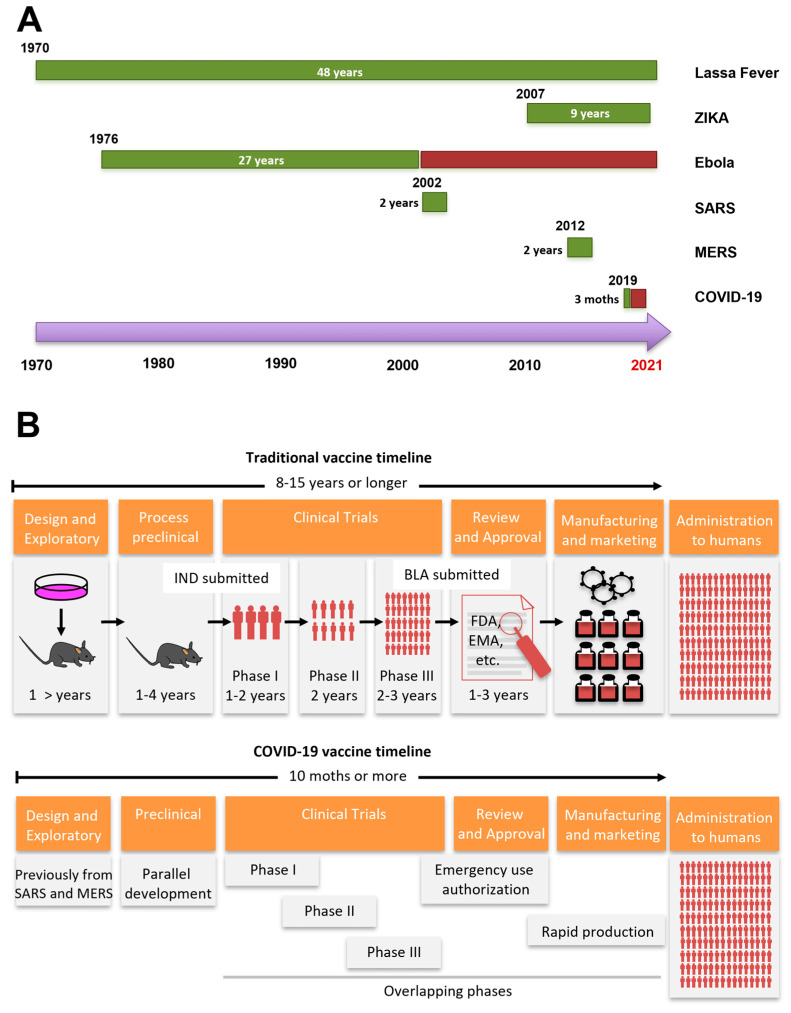
Recently emerging diseases vaccine and development timeline. (**A**) Date indicates the first major outbreak, (green) time to the first Phase I Clinical Trial. (Wine) indicates the time to get an approved or authorized Ebola and COVID-19 vaccine. (**B**) Comparison of traditional vaccine timeline for infectious diseases versus COVID-19 vaccine timeline.

Traditionally, vaccine development is a slow process and the timelines are generally measured in years or decades. An example is the Ebola vaccine that took approximately 27 years to develop. However, major advancements in recombinant genetic and messenger RNA (mRNA) technologies have been made, as well as knowledge acquired during the recent outbreaks of other human CoVs have contributed to the development of vaccines against COVID-19 in a shorter period of time (Figure 1A).

In the scientific area and health service communities, it is a true statement that a post-COVID-19 reality would be based on a robust immunization program. However, a significant hurdle to reaching this is that a traditional vaccine development program on average takes anywhere from 8 to 15 years to achieve regulatory approval before manufacturing and distribution (Figure 1B). Furthermore, the trend in past decades has been to require a growing and more complex number of clinical studies before approval. As a result, promising vaccine candidates that have established potential clinical proof-of-concept fail through the vaccine development pipeline [17].

There is a list of modern research tools that make it possible to develop vaccines against COVID-19 in a shorter time. This includes an unprecedented global response, the availability of genomic sequences in open sources, the set-up of complementary DNA (cDNA) reverse genetics methodology, and modern viral vectors, such as adenoviruses. Additionally, several COVID-19 vaccine candidates were developed rapidly due to prior knowledge from SARS-CoV-1 and MERS-CoV, overlapping phases 1, 2 and 3 clinical trials and rapid product development (Figure 1B).

## 3. Animal Models

Several animal models have been developed to examine SARS-CoV-1 and MERS-CoV infections, including cynomolgus monkeys, rhesus macaques, African green juvenile monkeys, squirrel monkeys, common marmosets, ferrets, hamsters, mice and camels [18,19,20,21,22]. All COVID-19 studies that use animal models require BSL-3 laboratories and high investments. Even with many animal model choices, mice are still the most used. Several mouse strains such as C57BL6 (B6), STAT1-/- and 129S Sv/Ev mice have been described to be susceptible to SARS-CoV-1 infection [23,24]. However, the most common models to date for SARS are older BALB/c mice (12–14-month) infected with a virus that been adapted to grow in mice (SARS-MA15) [25] and transgenic mice expressing the SARS-CoV-1 receptor human angiotensin-converting enzyme 2 (ACE2) on a C57BL/6 background (K18-hACE2). SARS-CoV-1 infections in these mice led to the development of clinical symptoms similar to the acute respiratory distress syndrome observed in humans, including death [26,27].

For in vivo studies of MERS-CoV, a transgenic mouse encoding the receptor human dipeptidyl peptidase-4 (DPP4) is generally used, which recapitulates viral replication with interstitial pneumonia [28].

To study COVID-19 in mice, two different strategies have been followed. The first has been to develop a mouse-adapted to SARS-CoV-2 to be able to utilize the mouse ACE2. To this end, sequential passages of SARS-CoV-2 in mouse lung tissue were performed [29,30]. The second strategy to make a mouse susceptible to the infection has been to express the human version of ACE2 in mice by generating a transgenic mouse model [31]. Both approaches lead to SARS-CoV-2 infection in mice and the development of severe disease. However, currently, no mouse model recapitulates all the different features of COVID-19 in humans. Furthermore, to establish a correlation closely between primate and human COVID-19 several nonhuman primates (NHP) include rhesus macaques, cynomolgus macaques and African green monkeys are studied [32,33]. A recent study identified Macaca mulatta as a good model for the study of SARS-CoV-2 infection and reproduce several human-like conditions [34]. Several studies development in NHP help to understand various aspects of COVID-19 disease as (airborne transmission, reinfection, inoculation and pathogenesis) [35,36,37,38].

Additionally, Syrian hamsters, Ferret, mink, cats, dogs, pigs, chickens, ducks, and fruit bats have also been used to study aspects of SARS-CoV-2 infection including transmission, ecology, and viral evolution [39,40].

## 4. Vaccine Platforms

**Figure 2:**
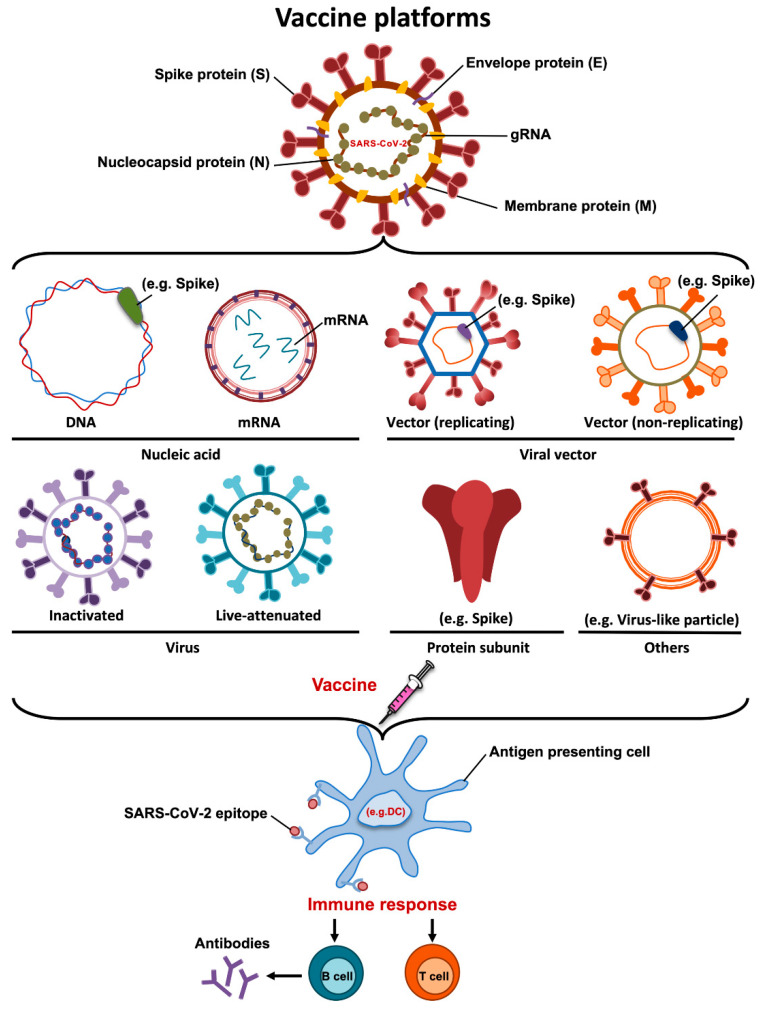
Illustration showing the several types of vaccine approaches currently being developed for COVID-19 disease.

An ideal vaccine needs to include a robust immune response with high neutralizing antibodies, T cell immunity and low or limited adverse events. Additionally, the protection should be conferred in two levels, preventing the manifestation of clinical disease and/or stopping viral transmission [14]. More than one hundred vaccine candidates, many based on different vaccine platforms, have been developed against SARS-CoV-2. These include DNA, mRNA, inactivated vaccines, viral vector-based vaccines, recombinant protein subunit vaccines and live attenuated vaccines (Figure 2). Interestingly, authorized or more advanced vaccine candidates include a combination of novel and more conventional approaches (Table 1). Here, we summarize the main vaccine platforms used for the generation of vaccine candidates against SARS-CoV-2.

### 4.1. DNA Vaccines

DNA vaccines are based on delivering a DNA plasmid that contains a gene encoding the antigen of interest. DNA vaccines induce long-lasting immunity and are very stable. The main advantages of DNA vaccines are their low production costs and the availability of efficient large-scale production. However, DNA vaccines often show relatively poor immunogenicity [41]. To date, ten SARS-CoV-2 DNA vaccines are under development including one in phase 3, but no DNA vaccines have been authorized for commercialization.

### 4.2. mRNA Vaccines

mRNA vaccines consist of mRNA that encodes the antigen of interest and are usually delivered into the cells via lipid nanoparticles. RNA vaccines have several advantages, including the generation of strong cellular and humoral immune responses, the absence of antivector immunity, and are easily scalable [42,43]. Furthermore, this approach is safe and effective because the mRNA carries a message but does not interact with the host genome. In contrast, the main disadvantages of mRNA vaccines are their low stability of RNA and the requirement of ultracold storage before distribution.

To date, 16 mRNA vaccines are being developed and 2 mRNA vaccines encoding SARS-CoV-2 S protein have been authorized for distribution (Pfizer-BioNTech and Moderna).

### 4.3. Inactivated Vaccines

Inactivated vaccines are one of the most conventional approaches for vaccine generation. In this case, vaccines are produced after growing the virus in cell culture, followed by physical or chemical inactivation methods. The main advantage of inactivated vaccines is that, due to the presence of the whole virus being administrated, more than one antigen is recognized by our immune system, leading to high immunogenicity [44]. However, the disadvantages of these vaccines include the difficulty of generating preparations of sufficient viral titer and the necessity of biosafety level 3 laboratories to generate the viral stocks. To date, there are sixteen inactivated whole-virus vaccine candidates in development, eight already in phase 3 and 2 vaccine candidates have been authorized, including CoronaVac, which is developed by Sinovac Biotech in China.

### 4.4. Live Attenuated Vaccines

Live attenuated vaccines are generally generated by either using a non-virulent strain or by the development of a weakened version of the virus. Virus attenuation can be achieved by using multiple strategies, including repeated passages in cultured cells and genetic modifications [45,46,47]. An important advantage is that live attenuated vaccines can be administered intranasally, closely mimicking a natural infection and inducing mucosal immune responses. In contrast, disadvantages of live attenuated vaccines include safety concerns, the requirement of extensive quality controls to ensure that the virus does not revert to a virulent form and that these vaccines cannot be used in elderly and immunocompromised populations [48]. To date, there are two live attenuated vaccine candidates against SARS-CoV-2 in evaluation.

### 4.5. Recombinant Protein Vaccines

Recombinant protein vaccines contain proteins derived from pathogens that promote the host immune response. Recombinant proteins can be produced by different expression systems, including bacterial, insect and mammalian cells. The main advantages of recombinant protein vaccines are that they can be produced in the absence of live viruses and that there is a significant experience in producing them. In general, whole or parts of the spike protein, including S1, S2, or RBD are the most frequent antigens used as candidate vaccines. However, recombinant proteins are not always easy to obtain and protein subunit vaccines are expensive to produce. To date, 31 projects are in development and five in phase 3.

### 4.6. Viral Vectors-Based Vaccines

Viral vectors-based vaccines are platforms using attenuated or replication-incompetent viruses as vectors encoding the antigen of interest. Viral vectors are characterized by eliciting strong immunogenicity and good stimulation of T and B cell responses. The main disadvantage is the presence of pre-existing immunity against the vector which could limit the effectiveness of these vaccines. Adenovirus, vaccinia, Newcastle disease virus, alphavirus and lentivirus are examples of viral vectors used to carry viral DNA fragments to promote immunity. Interestingly, to avoid the pre-existing immunity to some adenoviruses, a chimpanzee adenovirus (ChAdOx1 nCoV-19) is used as an alternative. Currently, seventeen vaccine candidates using viral vectors are being developed and four have been authorized.

## 5. Currently Authorized SARS-CoV-2 Vaccines

The landscape of vaccines to overcome the COVID-19 pandemic are based on several approaches, including (i) RNA vaccines, such as BNT162b2 (BioNTech, Fosun Pharma and Pfizer) as well as mRNA-1273 (Moderna); (ii) adenovirus-based vaccines, e.g., ChAdOx1 nCoV-19 (AstraZeneca/University of Oxford), Sputnik V (Gamaleya Research Institute), Ad26.COV2.S (Johnson and Johnson) or Ad5-nCoV (CanSino); (iii) inactivated virus, including BBIBP-CorV (Sinopharm) and CoronaVac (Sinovac); or (iv) protein subunits vaccines (Novavax), among others.

**Figure 3:**
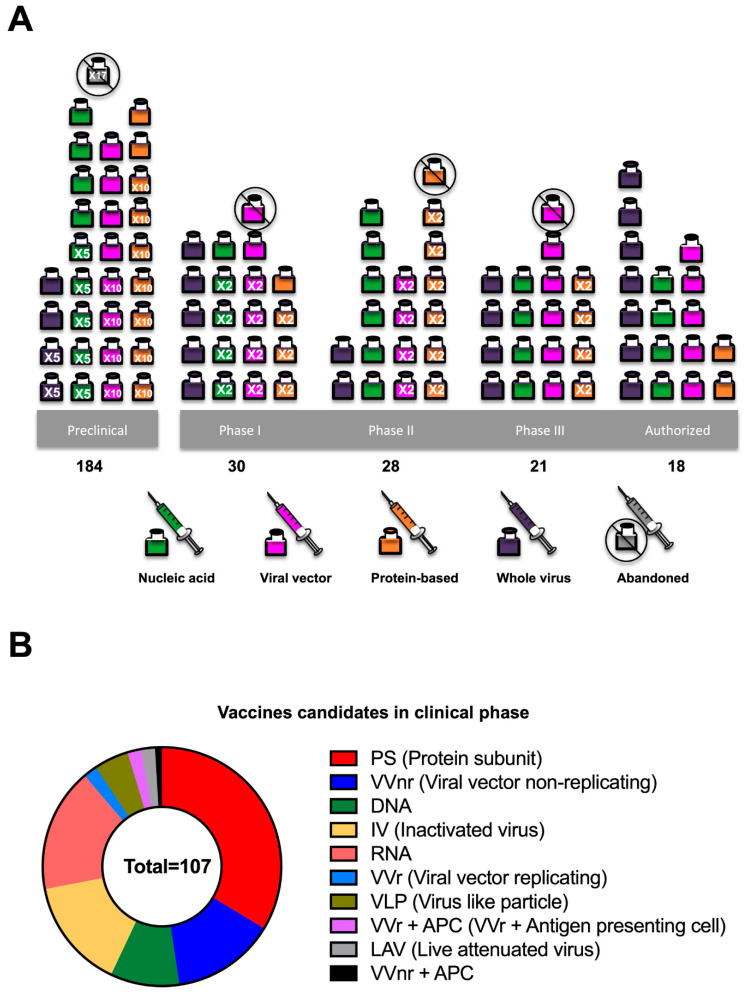
The COVID-19 candidate vaccine landscape. (**A**) Vaccines developed in preclinical and clinical phases vs vaccine platforms. (**B**) Vaccines candidates in the clinical phase. Based on WHO data [49].

As of 1 June, the list of vaccines authorized by the Food and Drug Administration (FDA) or the European Medicines Agency (EMA) is limited. However, the vaccines listed above have shown relevant results regarding efficacy in phase 3 clinical trials (Table 2). By 12 July, 107 and 184 vaccines are in clinical and preclinical development, respectively [49] (Figure 3).

In this review, the main results from clinical trials will be summarized and discussed to evaluate their effort to prevent either SARS-CoV-2 infection or to reduce the COVID-19 severity.

## 6. Acquired Immunity

The variety of vaccines have significantly shown robust immunogenicity, both in terms of antibody and the cell-mediated response. Starting with mRNA vaccines, two doses of the BNT126b2 vaccine [57,58] elicited a CD4+ T cell response in 94% of participants. Furthermore, the specific CD8+ T cell response was strong and appeared in 92% of participants. Antibody response also peaked at day 14 after the second dose, with 1.7 to 4.6-fold higher titers than convalescent serum. The second vaccine, mRNA-1273, [59,60] induced 5–6-fold higher titers of neutralizing antibodies 28 days after the first dose, with a 60–91% of seroconversion. Additionally, the seroconversion reached 100% 14 days after the second dose. However, the mRNA-1273 vaccine showed only modest results in CD8+ T cells response, as low levels were detected 14 days after the second dose.

Regarding adenovirus-based vaccines, the ChAdOx1 nCoV-19 vaccine [61] showed specific T-cell responses that reached their highest level on day 14. Anti-spike IgG responses increased by day 28 and improved with a boost. Antibody seroconversion increased up to 100% of participants after a single dose. The vaccination with one dose of the Ad26.COV2.S vaccine [62,63,64], resulted in 90% seroconversion by day 29 after administration. Additionally, robust CD4+ and CD8+ T cells responses were present on up to 83% of volunteers by day 15. The T cell responses were also kept against emergent SARS-CoV-2 variants [65]. Moreover, the combination of rAd26-S and rAd5-S [66] exhibited a seroconversion rate of 100% 42 days after vaccination and CD8+T cell response in all participants 14 days after the second dose. Alternatively, the sera collected was able to neutralize wild-type virus in 90% of volunteers 29 days after the first vaccine dose and peaked 100% 57 days after the second dose.

## 7. Efficacy against COVID-19 and Protection of the Elderly

Here, we summarize the data with a special emphasis on how to avoid severe COVID-19 and to protect the elderly, who are most vulnerable to COVID-19 [67].

Both mRNA vaccines, BNT162b2 and mRNA-1273, showed high efficacy on their first published phase 3 clinical trials, 95% [50] and 94% [52], respectively. However, although they enrolled more than 43,000 people (BNT162b2) and 30,000 people (mRNA-1273), a discrete amount of PCR-confirmed COVID-19 cases in vaccinated versus (vs.) placebo were identified (i.e., 8 vs. 162 in BNT162b2 and 11 vs. 185 in mRNA-1273). These numbers were decreased when data referred to severe COVID-19 7 days after the second dose of BNT162b2 or 14 days after the second dose of mRNA-1273: 1 vs. 4 (75% efficacy) and 0 vs. 30 (100% efficacy), respectively. Although no mass vaccination data has been published with mRNA-1273, in a clinical trial [51] that included almost 1.2 million people, half of them vaccinated with BNT162b2, detected a higher number of severe COVID-19 cases (55 vs. 174) and found a 92% effectiveness 7 days after the second dose, confirming results from the previous BNT162b2 study. Regarding efficacy with the elderly, BNT162b2 showed 95% (1 vs. 19) while mRNA-1273 did 86% (4 vs. 29) in people older than 65. Additionally, efficacy from BNT162b2 in the elderly was confirmed in the 1.2 million people study [51], even with one dose [68].

Adenovirus-based vaccines showed a variety of efficacy. In one phase 3 clinical trial, ChAdOx1 nCoV-19 [53] exhibited dose-dependent results, where half of the first dose increased the overall efficacy from 62% to 90% compared to two normal doses. The efficacy of this vaccine in preventing asymptomatic disease was 62% [69] and increased in preventing severe COVID-19 when doses were extended from 4 to 12 weeks: 55% to 81% [53]. Furthermore, although the ability of this vaccine to prevent symptomatic COVID-19 cases was demonstrated [53,54], only 3 out of almost 24,000 people were considered as severe COVID-19 cases and all of them were in the placebo group. This indicates that further studies are required to be able to compare its efficacy with the RNA vaccines. In the elderly, one dose of ChAdOx1 nCoV-19 was able to prevent hospitalizations [68]. Another adenovirus-based vaccine, Ad26.COV2.S showed an overall efficacy of 66% in preventing any COVID-19 cases 28 days after vaccination with a unique dose in phase 3 clinical trial [55]. This resulted in an increased avoidance of severe disease up to 85% for the same time point. The combination of rAd26-S and rAd5-S [56], however, showed efficacies of 91% against PCR-confirmed cases and 100% against severe COVID-19, both by the second dose. Both of these vaccines, Ad26.COV2.S, as well as the combination of rAd26-S and rAd5-S exhibited discrete cases of severe COVID-19 on the specified conditions, 39 and 20 people respectively, as well as ChAdOx1 nCoV-19. Although this indicated that they might prevent severe disease, a mass vaccination clinical trial is required to confirm these results.

## 8. Efficacy of Vaccines against the New SARS-CoV-2 Variants

The natural evolution of SARS-CoV-2 during its spreading around the world has implicated mutations in its genome that eventually led to the emergence of a variety of variants of different relevance [70,71,72]. Mutations in the S protein gene [73,74,75] are especially worrisome, as almost every available vaccine is based on the expression of the S protein with either mRNA- or adenoviral-based vaccines, as noted above. New variants including, B.1.1.7 (Alpha) identified in the UK, B.1.351 (Beta) in South Africa, P.1 (Gamma) in Brazil, and B.1.617.2 (Delta) in India have been associated with a higher risk for vaccination campaigns due to their increased transmissibility and potential vaccine evasion. In this review, we analyze several studies that have been performed to clarify whether the acquired vaccine-induced immune response leads to virus neutralization and protection.

The B.1.1.7 variant from the UK is thought to be up to 60% more transmissible [76] and harbors 7 substitutions in the S protein, N501Y, A570D, D614G, P681H, T716I, S982A, D1119H, and deletions of amino acids 69, 70 and 144 [77]. In vitro experiments have noted a modest reduction of neutralizing activity of sera collected from people vaccinated with ChAdOx1 nCoV-19 or BNT162b2 against B.1.1.7 [78]. However, another study showed no differences in neutralization activity from BNT162b2 vaccinated sera [79]. In addition, a randomized controlled trial showed a 70% efficacy of ChAdOx1 nCoV-19 vaccine for Alpha variant; and BNT162b2 effectiveness has been assessed as 87% [79]. Adding on, vaccination with mRNA-1273 induced neutralizing antibodies at the same level as infection, highlighting the likely protection of this vaccine against B.1.1.7 [80]. This suggests that vaccines overall protect the population despite the decrease of neutralization activity pointed by some in vitro studies.

The P.1 variant from Brazil includes 11 substitutions in S protein: L18F, T20N, P26S, D138Y, R190S, K417T, E484K, N501Y, D614G, H655Y, T1027I [81,82]. In vitro studies have shown that BNT162b2 elicited sera equally neutralized wild-type and a recombinant SARS-CoV-2 expressing an S protein with P.1 variant mutations, indicating an unlikely viral escape from vaccination [83]. The ChAdOx1 nCoV-19 or BNT162b2 vaccination sera showed in vitro a modest decrease in neutralization activity against the P.1 variant [84]. Interestingly, another in vitro study indicated that the sera collected from naturally infected people showed less neutralizing activity against the Gamma variant than those obtained from vaccinated with BNT162b2 or mRNA-1273 vaccines [85].

The B.1.351 variant from South Africa includes 8 changes in the S protein (D80A, D215G, K417N, E484K, N501Y, D614G, A701V) and residues 241 to 243 are deleted [86]. It has been reported that these mutations have allowed the virus to be more transmissible [87]. In vitro neutralization studies showed high resistance of Beta variant to antibodies elicited by ChAdOx1 nCoV-19. In addition, the efficacy of this vaccine against B.1.351 only reached 21% [88]. In contrast, Ad26.COV2.S vaccine showed an 82% of efficacy in preventing severe COVID-19 in a cohort with 94.5% cases of B.1.351 variant [55]. Similar results were found on mRNA vaccines when the BNT162b2 vaccine effectiveness studied in a cohort was 72% [89] and mRNA-1273 elicited sera showed significant neutralization despite reduced titers [90]. The fact that vaccination with BNT162b2 halved the cases and reduced severity in a COVID-19 outbreak indicates protection and diminished transmission [91].

The variants B.1.617 and B.1.618 from India have been also analyzed in neutralization experiments with pseudotyped lentiviruses expressing S protein with both variant’s sequences. Both convalescent sera and sera from vaccinated people with either BNT162b2 or mRNA-1273 showed a limited decrease when neutralizing them [92]. Additionally, BNT162b2 and ChAdOx nCoV-19 were effective in reducing SARS-CoV-2 infections and hospitalizations caused by B.1.167, although with a lower effect than those caused by the Alpha variant [93].

As shown before, the emergent variants of concern generally reduce vaccine-acquired neutralizing titers when serum is analyzed in vitro. However, to date, no complete vaccine-resistant variant of concern has been identified, particularly in people who received complete vaccination. Furthermore, mass vaccination campaigns reduce the spreading of SARS-CoV-2 and the emergence of new COVID-19 outbreaks.

New variants are likely to continue appearing in countries with a high cumulative incidence of COVID-19 due to the absence of actions against contagious or vaccination. Importantly, despite the coincidence that SARS-CoV-2 and population with partial immunity may lead to the emergence of escape variants, vaccination should not be but accelerated [94,95,96]. Furthermore, it is expected that countries whose economy grants access to mass vaccination become COVID-19-free regions, while others remain as COVID-19-endemic countries where the virus can freely evolve to escape variants.

EMA and FDA contemplate the feasibility that new adapted vaccines against escape variants have to be developed to address the emergence of potential escape variants of concern [97,98]. Data from non-clinical studies are limited or unnecessary when the manufacturer and producer are the same as the parental vaccine. However, FDA does encourage experiments with an appropriate animal model to test the new vaccine in a challenging study. Both institutions agree that new vaccines should demonstrate their immunogenicity and efficacy in clinical trials. Furthermore, the new vaccine may be considered a one-dose booster in the previously vaccinated population.

## 9. Adverse Events and Safety

In an optimal situation, an adverse event would be directly related to a vaccine using a specific laboratory test in the best accurate models. The problem with those tests is that they are not available for the most adverse events. Consequently, in most cases, events are only linked by their timing: a vaccine is administered and, at some point afterward, the person experiences the adverse effect. This makes it particularly challenging to establish causality between the medical problem and the vaccine, especially when the reaction occurs in the middle term after the immunization [99].

To establish the causal relationship of adverse events following immunization, several organizations, such as EMA, FDA and WHO conduct studies to determine the probability of having an adverse event after receiving the vaccine compared with the probability of having the same adverse effect in people who did not receive the vaccine. They also need to determine the mechanism that could have caused the reaction [99].

COVID-19 vaccines that are currently being administrated were strongly analyzed in clinical trials involving thousands of volunteers. Only after that, international organizations and governments authorized their use worldwide. The clinical trials are set up to clarify the points about efficacy and to assess the rate of quite common adverse effects [56,100,101,102,103,104] (Table 3), such as pain at the point of injection or nausea. It is important to emphasize that all the different vaccines authorized against COVID-19 are safe and effective for the general population and will help control the pandemic. However, clinical trials are not designed to detect extremely rare side effects, which might occur in fewer than one case per 10,000 vaccinations. As a result, very rare events, such as severe allergic reactions or thromboembolisms, are not reported until hundreds of millions of people have been vaccinated [99,105]. The main goal of these reports and the clinical challenge now is to work out which of these events are linked to the vaccine.

### 9.1. Anaphylaxis after COVID-19 Vaccination

In all cases, organizations such as CDC, FDA, and EMA teams are evaluating reports from vaccinated patients who experienced anaphylaxis, chronologically after getting the SARS-CoV-2 vaccine. This allergic reaction following COVID-19 immunization is a very rare adverse event corresponding to an estimated rate of approximately 2.5 cases per million based on EMA reports [105,106].

### 9.2. Thrombosis with Thrombocytopenia Syndrome (Tts)

During massive vaccination programs, concerns were about possible thromboses after immunization with the AstraZeneca vaccine. First, reports from the European Medicine Agency described thrombosis in four people immunized with one dose of the same batch of the vaccine, two of them were severe and one exitus [107].

This batch was retired from use. After that, one report from Denmark was released with one death case and vaccination was temporarily suspended to perform clinical investigation. This happened in other European countries [108].

Because of this, more attention was focused on thrombosis including cerebral venous sinus thrombosis (CVST). Rates of CVST are 15 cases per million each year according to recent studies [109]. CVST affects predominantly young to middle-aged people and women. These cases were rare and severe side effects take place 4–14 days after the first dose.

The thrombosis associated with the AstraZeneca vaccine was recently reported as thrombosis with thrombocytopenia syndrome (TTS). This rare condition could be associated with blood clots (thrombosis) and thrombocytopenia (low levels of blood platelets). Blood clots can take place in different parts of the organism, such as the brain (CVST) or in the abdomen. The mechanism that causes TTS is still not fully studied, but first studies point to the fact that is similar to heparin-induced thrombocytopenia (HIT), a rare immune reaction to treatments with heparin. The onset symptoms take place 4–26 days after vaccination.

At some point in different clinical trials, immunization plans were paused until a clinical investigation of this effect was performed. This rare condition presents an extensive thrombosis with thrombocytopenia and the development of autoantibodies against platelet-factor 4 (PF4) [110]. PF4 autoantibodies interact with the FcRγIIA receptor of platelets and contribute to aggregation. This strange condition has a similar onset of the classical HIT disorder after treatment with heparin. The interesting part is none of the affected vaccinated people were treated with heparin before this adverse effect. Recent studies revealed that COVID-19 could be associated with several autoimmune disorders with the expression of pathogenic autoantibodies and the new onset of autoimmune pathologies [111]. While COVID-19 immunization develops the synthesis of specific SARS-CoV-2-proteins, it is possible to produce PF4 autoantibody through molecular mimicry effect, due to activation of immune cells by vaccination [112].

Overall, the rate of TTS in vaccinated people is estimated to be about 6 cases per million. However, the rate is estimated to be 20–40 cases per million in those under 50 years of age. In fact, with 8 million doses administered, only 15 confirmed TTS cases have been reported as of April 2021 (7 per million in 18–49 years old and 0.9 cases per million in people with more than 50 years old) [113].

### 9.3. Reports of Death after COVID-19 Vaccination

Organizations such as the CDC and EMA use a vaccine adverse event reporting system (VAERS and PRAC) to closely monitor reports of death and adverse effects following COVID-19 vaccination. Something important to notice is that reports of deaths and adverse effects sent to these systems following vaccination do not necessarily mean that the death or adverse effect event and vaccination are related. In this line, the VAERS disclaimer specifically warns about using its data in the scientific literature [114,115]. In fact, those databases receive all the reports of any kind, even patients or relatives, if they were vaccinated. CDC must confirm all the cases and after that, establish the cause-effect relationship. At this time, less than 50% of reported cases had been confirmed and <0.1% had been established as vaccine-related [116].

On April 2021, the EMA team performed an analysis of 62 cases of CVST and 24 cases of splanchnic vein thrombosis reported in the EU pharmacovigilance database (EudraVigilance). On March 22, 2021, only 18 were exitus [117].

In Germany, since COVID-19 immunization started in December 2020, 22 deaths were reported concerning the vaccination. All cases had severe cardiovascular disease and other comorbidities [118]. It is crucial to note that to date these cases are not vaccination-related.

As expected, COVID-19 deaths also occur after immunization as, although vaccination has a high efficacy rate in preventing severe pathology and death from COVID-19, vaccines are not always 100% effective. Furthermore, contracting COVID-19 is still possible even after receiving a second dose [50]. In addition, nasopharyngeal swabs during autopsies, to detect viral RNA, should also be contemplated if there is evidence of pneumonia [118].

### 9.4. Myiocarditis Following COVID-19 Vaccination

Myocarditis is the condition of inflammation of the middle layer of the heart tissue [119]. On June 23, 2021, the CDC included myocarditis and pericarditis as a benefit-risk assessment of COVID-19 mRNA vaccination especially in the young population [120,121]

Since April this year, more than a thousand reports to VAERS of cases of myocarditis and pericarditis following mRNA COVID-19 vaccination [122]. All patients had a confirmed diagnosis of myocarditis with no infectious, ischemic, or autoimmune etiologies. Diagnoses were reviewed by all protocols and followed the CDC definition criteria in cases of potential myocarditis. Based on different observational studies, all cases were resolved within 1 week of cardiac onset. Several patients continued having chest discomfort 2 months later [123].

In the context of COVID-19 vaccination clinical trials, adverse heart effects of any kind were reported in <0.1% of patients and rates were similar in vaccine receivers compared with those with placebo [124].

Actual rates of myocarditis in the general population receiving vaccination are variable and still being determined by CDC. The actual estimate of incidence is 22 cases per 100,000 people [125]. CDC and FDA have confirmed 699 cases of myocarditis/pericarditis. These clinical teams are performing an investigation of the reports to confirm if there is a relationship to SARS-CoV-2 vaccination [122].

### 9.5. Antibody-Dependent Enhancement in COVID-19

Information obtained from other respiratory coronaviruses is crucial to study the hypothesis that SARS-CoV-2 antibodies could lead to a more severe COVID-19 disease through antibody-dependent enhancement (ADE) [126].

ADE is a condition that affects the clinical course of several viral infections, such as Dengue virus [127] and other respiratory infections, including measles [128] or the Respiratory Syncytial Virus (RSV) [129,130]. In this context, ADE can be classified in an extensive category termed enhanced respiratory disease (ERD), with non-antibody-based mechanisms such as cytokine storm and cell-mediated immune pathologies [126]. Furthermore, ADE and ERD have been reported for other viral infections in the Coronavirus family, including SARS and MERS. The molecular pathways by which ADE could contribute to COVID-19 pathogenicity are currently being extensively studied.

ADE could take place when non-neutralizing or sub-neutralizing antibodies bind to viral antigens without neutralizing the virus and transport and internalize virions directly to macrophages, which then become productively infected [127]. Interestingly, for example, secondary infections with heterologous strains could lead to more severe dengue disease. This is due to high concentrations of antibodies against different dengue serotypes that are cross-reactive but non-neutralizing [131]. Furthermore and in a respiratory context, Fc-mediated antibody effector can lead to a severe clinical course by developing strong immune pathways that result in noticeable lung disease [132].

In the context of COVID-19 vaccine safety, those with high levels of the theoretical potential risk of ADE/ERD are vaccines based on inactivated viruses, that usually are developed with non-neutralizing antigen targets [126]. Consequently, potential risks of ADE from vaccines may be reduced by the administration of potent neutralizing antibodies in high doses.

Fortunately, to date, no signs of ADE have been described during the preclinical animal studies, during the human trials or mass vaccination campaigns worldwide with more than 3 billion doses of the different authorized vaccines against COVID-19 administered, even with the different and potentially dangerous variants identified circulating worldwide.

## 10. Concluding Remarks

This review offers a broad landscape of COVID-19 vaccines, with special attention to FDA or EMA authorized ones. The new insights about the design and development of COVID-19 vaccines, the acquired immunity, efficacy, and undesirable side effects were summarized and compared to better understand the state-of-the-art vaccines. In addition, key information regarding different coronavirus species was provided to give context to the COVID-19 pandemic.

CoVs are examples of zoonotic-emerging viruses that frequently cross the species barrier and emerge in humans. To date, seven human coronaviruses cause mild upper or severe respiratory syndrome. In the last 20 years, three highly pathogenic coronaviruses emerged SARS-CoV-1 [133], MERS-CoV [134] and SARS-CoV-2 [135]. Therefore, SARS-CoV-2 may not be the last coronavirus to cause a global pandemic.

The emergence of SARS-CoV-2 impacts the world in several aspects, such as public health, mental health, and economics. Therefore, collaborative research groups around the world were focused on prioritizing COVID-19 studies, as vaccine design and drug candidates.

However, despite mass vaccination programs are underway in many countries all over the world, the end of the pandemic is not clear yet. The emergence of new variants is of concern and the growing vaccine inequity in some countries could compromise vaccine effectiveness. Therefore, no one is safe, unless everyone is safe. For this reason, the WHO, the Coalition for Epidemic Preparedness Innovations (CEPI) and the Global Alliance for Vaccines and Immunizations (GAVI) launched the COVID-19 Vaccines Global Access (COVAX) program [136,137,138].

Their objective is to accelerate the development and manufacture of COVID-19 vaccines. COVAX offers doses for at least 20% of countries’ populations to end the acute phase of the pandemic. Several countries with a high percentage of vaccination reaffirm that vaccines are the most effective ways to control and stop the spread of the pandemic of COVID-19 [51].

A broad multiplatform of vaccines has been described here, from mRNA to adenovirus-based vaccines. It is worth noting that it took less than a year to start vaccination campaigns out of clinical trials. Fortunately, they have shown very promising results from the beginning, with around 95% efficacies in phase 3 clinical trials [50,52]. This goal could never have been achieved without the prior relevant knowledge obtained about coronaviruses and vaccine development by that date. It includes the identification of SARS-CoV-1 ACE2-dependent cell entry [139,140], or the immunological response to SARS-CoV-1 proteins [141], which focused the studies on S protein.

## Figures and Tables

**Table 1:** Type of vaccine candidates with developers and stage of clinical evaluation.

Type of Candidate Vaccine	Developer (Some Examples)	Stage of Clinical Evaluation
DNA	Zydus Cadila Inovio Pharmaceuticals + International Vaccine Institute + Advaccine (Suzhou) Biopharmaceutical AnGes + Takara Bio + Osaka University	CTRI/2020/07/026352 (phase 2/3) NCT04642638 (phase 2/3) NCT04655625 (phase 3)
mRNA	BioNTech/Fosun, Pharma/Pfizer (BNT162b2) Moderna (mRNA-1273)	NCT04368728 (Authorized) NCT04760132 (Authorized)
Inactivated vaccine	Sinovac Research and Development Co, Ltd. Sinopharm + China National Biotec Group Co + Beijing Institute of Biological Products	NCT04756830 (Authorized) NCT04863638 (Authorized)
Live attenuated vaccine	Codagenix/Serum Institute of India Meissa Vaccines, Ins.	NCT04619628 (Phase 1) NCT04798001 (Phase 1)
Recombinant protein subunit vaccine	Novavax Sanofi Pasteur + GSK Instituto Finlay de Vacunas Federal Budgetary Research Institution State Research Center of Virology and Biotechnology “vector” Center for Genetic Engineering and Biotechnology (CIGB)	NCT04611802 (phase 3) PACTR202011523101903 (phase 3) RPCEC00000354 (phase 3) NCT04780035 (phase 3) RPCEC00000359
Viral vectors-based vaccines	AstraZeneca + University of Oxford CanSino Biological Ins./Beijing Institute of Biotechnology Gamaleya Research Institute; Health Ministry of the Russian Federation Janssen Pharmaceutical	NCT04760132 (Authorized) NCT045226990 (Authorized) NCT04530396 (Authorized) NCT04505722 (Authorized)

**Table 2:** Efficacies of main authorized vaccines in COVID-19 prevention.

Developer	Trade Name	Vaccine	Efficacy Preventing PCR-Confirmed COVID-19	Efficacy Preventing Severe COVID-19	References
BioNTech, Fosun Pharma and Pfizer	Comirnaty	BNT162b2	94–95%	75%	[50,51]
Moderna	Moderna COVID-19 Vaccine	mRNA-1273	94%	100%	[52]
University of Oxford and AstraZeneca	Vaxzevria	ChAdOx1 nCoV-19	60–90%	100%	[53,54]
Janssen (Johnson and Johnson)	Janssen COVID-19 Vaccine	Ad26.COV2.S	66–67%	77–85%	[55]
Gamaleya Research Institute	Sputnik V	rAd26-S and rAd5-S	91%	100%	[56]

**Table 3:** Common adverse effects.

Adverse Effect	Frequency (Pfizer-BioNTech)	Frequency (Moderna)	Frequency (University of Oxford/AstraZeneca)	Frequency (Sputnik-V)	Frequency (Janssen)
Fever	10.9%	91.6%	8.2%	2.2%	12.8%
Pain injection-site	66.1%	62.8%	71%	5%	58.6%
Fatigue	50.5%	67.6%	21.1%	2.5%	43.8%
Headache	39%	62.8%	22.8%	2.9%	44.4%
Muscle pain	28.7%	6.1%	7%	0.9%	39.1%
Nausea	0.7%	21.3%	5.7%	0.7%	15.5%
Chills	22.7%	48.3%	14.7%	0.4%	7%

## Data Availability

Not applicable.
